# Unusual cause of small bowel obstruction: mesodiverticular band of Meckel’s diverticulum demonstrated by CT

**DOI:** 10.1259/bjrcr.20150255

**Published:** 2016-10-25

**Authors:** Cisel Yazgan, Tolga Sahin, Mahir Ozmen

**Affiliations:** ^1^Department of Radiology, Hacettepe University Faculty of Medicine, Ankara, Turkey; ^2^Department of Surgery, Hacettepe University Faculty of Medicine, Ankara, Turkey

## Abstract

Small bowel obstruction resulting from the mesodiverticular band is a rare complication of Meckel’s diverticulum and usually presents a diagnostic challenge. We present a case of small bowel obstruction due to the mesodiverticular band of Meckel’s diverticulum with CT scan findings.

## Summary

Meckel’s diverticulum is the most common congenital anomaly of the gastrointestinal tract that is caused by incomplete closure of the omphalomesenteric duct.^1,2^ Although it is usually detected incidentally during a surgical procedure for another indication, 4% of the patients with Meckel’s diverticulum may present with life-threatening complications such as haemorrhage, intussusceptions, small bowel obstruction, diverticulitis and perforation.^3,4^ Small bowel obstruction is the most prevalent complication in adults with Meckel’s diverticulum, accounting for one-third of all complicated cases.^5^ It is caused by various mechanisms, including intussusception of an inverted Meckel’s diverticulum, volvulus, Littre’s hernia and internal herniation of the small bowel underneath the mesodiverticular band.^6,7^ The purpose of this case report is to show the CT features of mesodiverticular band of Meckel’s diverticulum associated with small bowel obstruction and to emphasize the serious nature of this condition if not diagnosed and treated promptly.

## Clinical presentation

A 35-year-old male was admitted to the emergency department with abdominal pain, vomiting and nausea. His past medical history was unremarkable. On physical examination, the abdomen was distended. Laboratory tests performed showed elevated levels of white blood cell count (14.5 × 10³ cm^−3^) and C-reactive protein (16.1 mg dl^−1^).

## Imaging 

**Figure 1.**
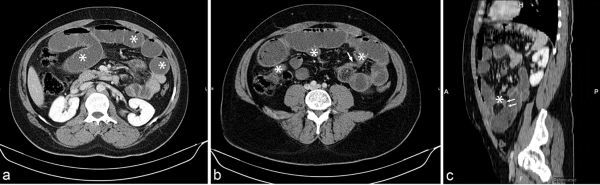
Contrast-enhanced** a**xial CT image (a) demonstrates dilated small bowel loops (asterisks) with multiple air–fluid levels. Axial CT image obtained at a lower level and sagittal image (b, c) show the fibrous band at the transition point (arrows). The collapsed distal ileum (asterisks in b, c) is also seen.

**Figure 2.**
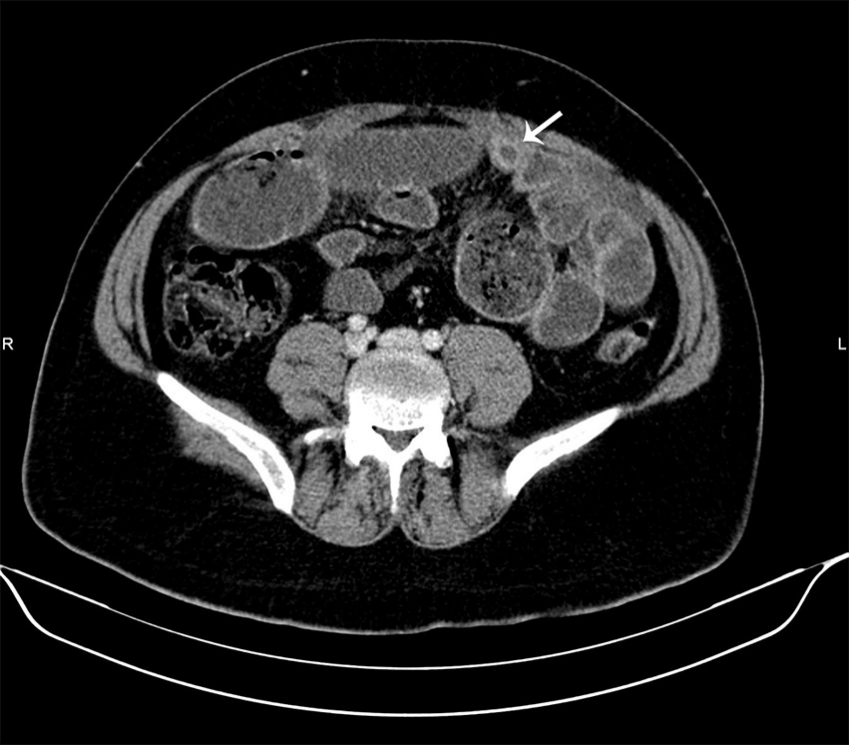
An axial CT image reveals a fluid-filled tubular structure in the lower abdomen (arrow).

**Figure 3.**
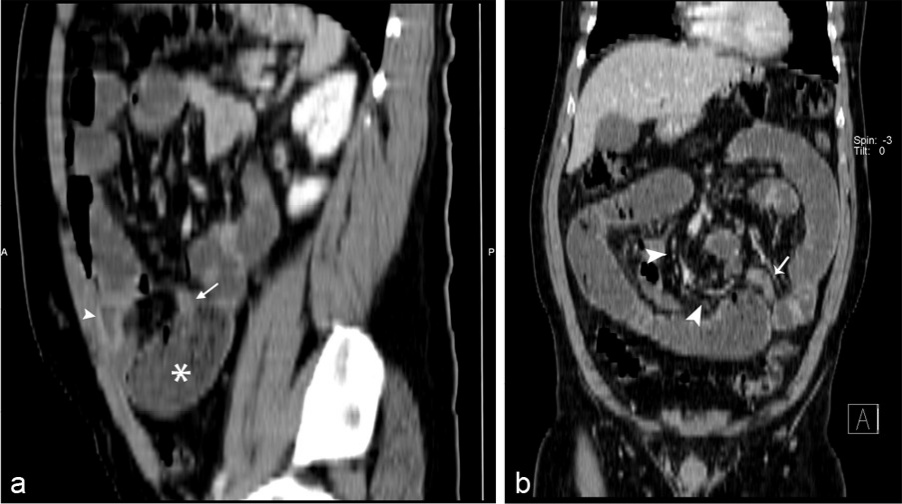
Sagittal reformatted image (a) shows a fibrous band (arrow) with dilated bowel loop (asterisk). The Meckel’s diverticulum (arrowhead) is also seen. Coronal reformatted image (b) illustrates converging mesenteric vessels (arrowheads), consistent with internal hernia and mesodiverticular band (arrow).

Plain abdominal X-ray revealed air–fluid levels in the small bowel. Contrast-enhanced CT scan showed markedly dilated loops of the middle and distal small bowel with collapsed distal ileum (Figure 1a,b). In the lower quadrant, there was a 4 cm blind-ending tubular structure containing fluid with a thickened wall in continuity with the small bowel. The structure was deemed to be a Meckel’s diverticulum (Figure 2a,b). A band-like lesion was demonstrated with multiplanar reconstructions in the adjacent mesentery (Figure 3).

## Treatment and outcomes

Considering the diagnosis of Meckel’s diverticulum with strangulated internal hernia, an emergency laparotomy was performed. As the small bowel loops were found to be markedly dilated, the bowel was explored from the Trietz’s ligament to the ileocaecal valve. There was a fibrous band at the tip of the diverticulum extending to the mesentery of the proximal bowel. The herniated ileal segments showed evidence of ischaemia. Segmental resection and primary end-to-end anastomosis were performed. The patient was discharged without any complications 4 days later.

## Discussion

Meckel’s diverticulum is a remnant of the omphalomesenteric duct, which connects the yolk sac with the developing midgut in fetal life. It normally regresses by the seventh to eighth week of gestation. However, incomplete involution of the omphalomesenteric duct results in various congenital anomalies such as omphalomesenteric duct cyst, umblicoileal fistula, umbilical sinus and Meckel’s diverticulum. The diverticulum usually occurs within 100 cm of the ileocaecal valve at the antimesenteric border of the ileum. The arterial supply comes from ileocolic branches of the superior mesenteric artery. Rarely, a persistent vitelline artery would supply the Meckel’s diverticulum through the mesodiverticular band.^8,9^ This embryologic band extending from the adjacent mesentery to the tip of the diverticulum creates a bridge through which bowel loops may be herniated and obstructed.^10^

Acute intestinal obstruction is one of the most frequently encountered clinical entities in the emergency departments. The most common causes are adhesions and neoplasms, while internal hernias are responsible for only 0.05–4.1% of the cases with intestinal obstruction.^11,12^ Early diagnosis and treatment are extremely crucial, as surgery delayed by more than 36 hours increases the mortality rate from 8% to 25% in patients with complications due to strangulation.^13^ Treatment of small bowel obstructions depends on the cause of the obstruction and the presence of intestinal strangulation.

Intestinal obstruction owing to mesodiverticular band has been reported in patients with Meckel’s diverticulum.^14–16,17,18^ In addition, Vork et al^19^ have pointed out the high mortality rate of Meckel’s diverticulum combined with mesodiverticular band and the importance of immediate surgery. In this setting, internal herniation of the small bowel loops underneath the mesodiverticular band is the main mechanism for bowel obstruction, as seen in the presented case. Patients with intestinal obstruction by congenital bands require surgical intervention and band resection.^3^ Therefore, pre-operative imaging plays a crucial role, both in preventing fatal complications and in determining accurate management. However, it is a challenge to make a correct diagnosis preoperatively of Meckel’s diverticulum complicated by small bowel obstruction.

Imaging features of the mesodiverticular band of the Meckel’s diverticulum have been published.^15,16^ Sun et al^20^ demonstrated the mesodiverticular band as a hyperechoic line in their cases and highlighted the feasibility of high frequency ultrasonography, particularly in children, in revealing congenital bands. However, they emphasized that CT is superior to ultrasonography in detecting the aetiology of small bowel obstruction accurately in adult or obese patients.

It is difficult to distinguish an uncomplicated Meckel’s diverticulum from a normal small bowel on a CT scan. However, Meckel’s diverticulum manifests as a blind-ending tubular segment or diverticular sac containing fluid. In a previous study, authors have reported that the majority of Meckel’s diverticula in their series are located at or near midline.^21^ In our case, the distal ileum was identified as a transition zone, with collapsed distal loops on pre-operative CT. A bridge or band-like lesion extending into the adjacent mesentery was demonstrated by coronal and sagittal reformatted images. Adhesion was excluded, as our patient had not undergone any abdominal surgery previously.

We have reported a mesodiverticular band of Meckel’s diverticulum leading to small bowel obstruction. Although it is hard to establish a pre-operative diagnosis, several CT features may suggest the diagnosis. First, fluid–air filled a blind-ending pouch arising from the antimesenteric side of the distal ileum, strongly suggesting Meckel’s diverticulum. Second, a mesodiverticular band can be considered by the presence of a bridge or band-like lesion connecting the tip of the Meckel’s diverticulum to the root of the mesentery. Multiplanar reformatted images may increase the diagnostic confidence in detecting fibrous bands. Third, characteristic CT findings of internal hernia, including converging mesenteric vessels or bowel loops, may support the appropriate diagnosis of mesodiverticular band in the setting of Meckel’s diverticulum.

In summary, the mesodiverticular band of Meckel’s diverticulum causing mechanical small bowel obstruction is a rare complication of this congenital anomaly. It should be considered in the differential diagnosis of a small bowel obstruction, especially in young patients with no prior history of abdominal surgery. CT is a very useful diagnostic tool in the diagnosis of Meckel’s diverticulum complicated by small bowel obstruction.

## Learning points

1. Complication of a Meckel’s diverticulum should be considered in the differential diagnosis of small bowel obstruction, particularly in patients with low probability for adhesive obstruction.
2. Mesodiverticular band is a rare cause of small bowel obstruction in patients with Meckel’s diverticulum and it is difficult to diagnose it preoperatively.
3. Recognizing the key CT features of the mesodiverticular band of Meckel’s diverticulum is important to make the correct diagnosis preoperatively and prevent fatal complications.

## Consent

Written informed consent for the case to be published (including images, case history and data) was obtained from the patient(s) for publication of this case report.
